# Gender differences in the prevalence of low back pain associated with sports activities in children and adolescents: a six-year annual survey of a birth cohort in Niigata City, Japan

**DOI:** 10.1186/s12891-019-2707-9

**Published:** 2019-07-13

**Authors:** Ren Kikuchi, Toru Hirano, Kei Watanabe, Atsuki Sano, Tsuyoshi Sato, Takui Ito, Naoto Endo, Naohito Tanabe

**Affiliations:** 1 Department of Orthopedic Surgery, Niigata Rosai Hospital, 12-7-1 touuntyou, Jouetsu City, Niigata Japan; 2 Division of Orthopedic Surgery, Department of Regenerative and Transplant Medicine, Niigata University Graduate School of Medical and Dental Sciences, 1-757 asahimachidori, chuoku, Niigata City, Niigata Japan; 3 Department of Orthopedic Surgery, Tsuruoka Municipal Shonai Hospital, 4-20 izumityou, Tsuruoka City, Yamagata Japan; 4 Department of Orthopedic Surgery, Niigata Prefectural Shibata Hospital, 1-2-8 hontyou, Shibata City, Niigata Japan; 5 Department of Orthopedic surgery, Nagata clinic, 4-1205 nagata, chuoku, Niigata City, Niigata Japan; 6 Department of Health and Nutrition, University of Niigata Prefecture, 471 ebigase, higashiku, Niigata City, Niigata Japan

**Keywords:** Gender differences, Low back pain, Children, Adolescents, Sport

## Abstract

**Background:**

This study was conducted to determine gender differences in the relationship between extracurricular sports activities (ECSA) and low back pain (LBP) in children and adolescents.

**Methods:**

In a cohort analysis of a 6-year birth cohort annual survey, students were followed from the fourth to sixth grades of elementary school (E4–E6; 9–12 years old) through the first to third grades of junior high school (J1–J3; 12–15 years old). All students completed annual questionnaires on ECSA and LBP. The odds ratio (OR) and 95% confidence interval (CI) were calculated to assess the association strength between ECSA and LBP. We also calculated the population attributable fraction (PAF), which was defined as the proportion of students with ECSA-related LBP among all students with LBP.

**Results:**

ECSA was significantly associated with LBP only in grade J3 among boys (OR: 2.00, 95% CI: 1.47–2.71). On the other hand, among girls, ECSA was significantly associated with LBP in grades E5 (OR: 1.48, 95% CI: 1.00–2.20), E6 (OR: 1.91, 95% CI: 1.33–2.75), and J3 (OR: 1.81, 95% CI: 1.26–2.61). Among boys, PAF was similar in all grades (range, 10–16%), whereas among girls, the PAF varied (− 11 to 29%) and was significantly higher in girls than in boys in grades E5 (19.0% vs. 1.1%, *P* < 0.01) and E6 (28.8% vs. 12.8%, *P* < 0.01).

**Conclusions:**

Although there was a link between ECSA and LBP in both boys and girls, girls were more susceptible to ECSA-related LBP, especially in grades E5 and E6.

## Background

In recent years, there has been an increase in the number of non-specific low back pain (LBP) cases reported among children and adolescents [1]. Factors such as age [2–4], gender [5], physical activity [2, 6], back strength [2], leg flexibility [7], physical posture [8], family history [9], mental health [9, 10], smoking [7] and television viewing time [11] are potential risk factors for LBP in this age group.

In addition, sports activity has also been suggested to be related to LBP in children and adolescents [12]. Although there have been some reports showing an association among LBP, sports activity and gender differences [13–17], no study has investigated these relationships for every year in children and adolescents for a certain period of time.

In 2005, we conducted a large-scale cross-sectional survey of all elementary school students in grades 4–6 (E4–E6; ages 9–10 years old (yo), 10–11, and 11–12 yo, respectively, 21,893 students) and all junior high school students in grades 1–3 (J1–J3; 12–13, 13–14, and 14–15 yo, respectively, 21,737 students) in Niigata City(at longitude 139° east and latitude 37° north, located on the west coast of Japan with an area 726.45 km^2^ and a population of 811,901 as of October 1, 2010), Japan. We found that the lifetime prevalence of LBP was significantly higher among students who participated in extracurricular sports activities (ECSA) than among those who did not, and students with a history of LBP spent more time on ECSA [18]. However, our cross-sectional study was unable to determine whether these results were unique to each grade or representative of a continuum.

In order to obtain more detailed information about LBP in children and adolescents, we conducted a 6-year birth cohort analysis of students who were in grade E4 at the time of the initial survey. The birth-cohort analysis based on annually conducted cross-sectional surveys revealed that participation in ECSA correlated with LBP in grades E6 and J3, when boys and girls were analyzed together [19]. However, boys and girls develop differently in many respects between the ages of 9 and 15 years. Therefore, the impact of gender on the relationship between ECSA and LBP in this age group must be determined.

## Methods

### Setting and participants

A single-birth cohort of students in Niigata City, Japan was followed for 6 years, beginning in 2005 when the students were in grade E4 (baseline) and ending in 2010 when the students were in grade J3. In Niigata City, there were 110 elementary schools in 2005, 114 elementary schools in 2006 and 2007, and 62 junior high schools in 2008–2010. The increase in the number of elementary schools in 2006 was due to the merging of a neighboring town into Niigata City in October 2005.

From 2005 to 2010, 107, 112, and 109 elementary schools and 57, 60, and 60 junior high schools participated in our survey each year. We distributed questionnaires to 6,969, 7,266, 7,269, 6,943, 7,042, and 7424 students from 2005 to 2010, respectively, and received responses from 4,597 (66.0%), 5,449 (75.0%), 5,408 (74.4%), 5,754 (82.9%), 5,588 (79.4%), and 5,800 (78.1%) students, respectively. Of those respondents, 4,451 (96.8%), 5,319 (97.6%), 5,273 (97.5%), 5,599 (97.3%), 5,421 (97.0%), and 5,366 (92.0%) gave valid responses to all questions on gender, present LBP, and ECSA.

### Data collection

Basic information, such as the name of the school, grade, and gender, was anonymously collected for each student. Multiple choice questions, which were answered voluntarily and were obtained by verbal consent, were used to obtain information about present LBP and ECSAs (including musical activities but not school physical education classes). Teachers were asked to notify guardians that this questionnaire was voluntary. This information was also orally communicated to students before questionnaires were distributed. For LBP, the question was “Do you have any pain in your lower back now?” (Yes/No). For ECSA, the questions were “Excluding physical education classes in school, do you participate in sports?” (Yes/No) and "If yes, “What sports do you play?” We also asked how many days per week and how long (minutes per day) the students participated in ECSAs. The specific details of the questionnaire have been presented in the previous publications [18–21].

The same questionnaire was administered annually in the fall. Elementary school students completed the questionnaire at home together with their parents or guardians, whereas junior high school students were allowed to complete the questionnaire by themselves. All questionnaires were later collected at school. Institutional review board approval for this study was granted by the Niigata City Board of Education.

### Definitions

In this study, students who answered yes to “Do you have any pain in your lower back now?” were defined as having LBP. In the Japanese school system, students spend 6 years in elementary school (grades E1–E6) and 3 years in junior high school (grades J1–J3).

### Outcome measures

The outcome measures in our study were the number and percentage of students participating in ECSA, point prevalence of LBP, odds ratio (OR) and population attributable fraction (PAF) for ECSA-related LBP [22], and average amount of time spent on ECSA (ECSA group only). These measures were evaluated in both boys and girls in each grade. The PAF was defined as the proportion of students with ECSA-related LBP among all students with LBP and was calculated as follows: (T - N × P0) ÷ T × 100, which is equal to (Ptotal - P0) ÷ Ptotal × 100, where T is the total number of students with LBP, N is the total number of students, P0 is the prevalence of LBP in the non-ECSA group, and Ptotal is the total prevalence of LBP. Although the PAF is usually used to determine the incidence of a disease, we used it to estimate the proportion of LBP associated with ECSA.

### Statistical analysis

Categorical variables and continuous variables were compared between the two groups using the chi-square test and Student’s t-test, respectively. And a Tukey-Kramer test was used for the comparison of the amount of time spent on ECSA between the grades. All statistical tests except for Student’s t-test and the Tukey-Kramer test were conducted using Excel 2007 (Microsoft Corp., Redmond, WA, USA). Student’s t-test and the Tukey-Kramer test were performed using SPSS 14.0 software for Windows (SPSS Inc., Chicago, IL, USA). Two-tailed *P* values < 0.05 were considered significant.

## Results

### Participation in ECSA in each grade

Regardless of gender, the percentage of students who participated in ECSA was approximately the same in grades E4 to E6 (Tables 1, 2). The percentage increased in grade J1 and considerably decreased in grade J3.

**Table 1:** Point prevalence of low back pain and extracurricular sports activities among boys by grade

Variable	E4	E5	E6	J1	J2	J3
ECSA (−)						
n (%)	607 (28.0)	701 (27.0)	883 (34.3)	377 (13.6)	439 (16.5)	2186 (83.9)
LBP (+) /LBP (−)	15/592	23/678	42/841	22/355	35/404	180/2006
LBP (+) /n (%)	2.5	3.3	4.8	5.8	8.0	8.2
ECSA (+)						
n (%)	1564 (72.0)	1891 (73.0)	1684 (65.6)	2394 (86.4)	2220 (83.5)	421 (16.1)
LBP (+) /LBP (−)	44/1520	63/1828	98/1586	171/2223	215/2005	64/357
LBP (+) /n (%)	2.8	3.3	5.8	7.1	9.7	15.2
Odds ratio	1.14	1.02	1.24	1.24	1.24	2.00
(95% CI)	(0.63–2.07)	(0.63–1.65)	(0.85–1.79)	(0.79–1.96)	(0.85–1.80)	(1.47–2.71)**
PAF (%)	9.1*	1.1	12.8	16.2	15.2	12.0

*Abbreviations*: *ECSA* extracurricular sports activities, *n* number of students, *LBP* low back pain, *PAF* population attributable fraction, *CI* confidence interval. E4–E6 and J1–J3 refer to elementary school grades 4–6 and junior high school grades 1–3, respectively. The PAF among boys was compared with that among girls. *P < 0.05, **P < 0.01

**Table 2:** Point prevalence of low back pain and extracurricular sports activities among girls by grade

Variable	E4	E5	E6	J1	J2	J3
ECSA (−)						
n (%)	1126 (49.4)	1322 (48.5)	1422 (52.5)	660 (23.2)	746 (27.0)	2437 (89.3)
LBP (+) /LBP (−)	35/1091	42/1280	49/1373	42/618	55/691	196/2241
LBP (+) /n (%)	3.1	3.2	3.4	6.4	7.4	8.0
ECSA (+)						
n (%)	1154 (50.6)	1405 (51.5)	1284 (47.5)	2188 (76.8)	2016 (73.0)	292 (10.7)
LBP (+) /LBP (−)	29/1125	65/1340	82/1202	163/2005	180/1836	40/252
LBP (+) /n (%)	2.5	4.6	6.4	7.5	8.9	13.7
Odds ratio	0.80	1.48	1.91	1.20	1.23	1.81
(95% CI)	(0.49–1.32)	(1.00–2.20)†	(1.33–2.75)**	(0.84–1.70)	(0.90–1.69)	(1.26–2.61)**
PAF (%)	−10.7	19.0**	28.8**	12.2	13.3	7.0

*Abbreviations*: *ECSA* extracurricular sports activities, *n* number of students, *LBP* low back pain, *PAF* population attributable fraction, *CI* confidence interval. E4–E6 and J1–J3 refer to elementary school grades 4–6 and junior high school grades 1–3, respectively. The PAF among girls was compared with that among boys. ^†^*P* = 0.05, **P < 0.01

### Amount of time spent on ECSA

**Fig. 1:**
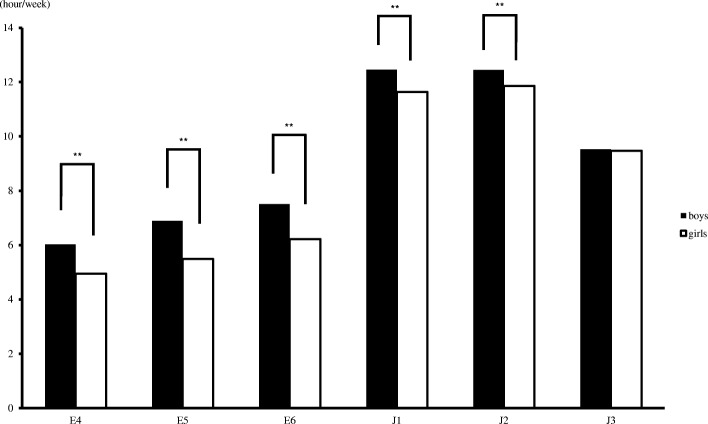
Differences in the amount of time spent on extracurricular sports activities based on gender

Compared with girls in the same grades, boys who participated in ECSA spent significantly more time doing so per week in grades E4 to J2 (*P* < 0.01) (Fig. 1). The amount of time spent on ECSA in both genders significantly increased in J1 compared with E6 (*P* < 0.01).

### Prevalence of LBP in students with and without participation in ECSA in each grade

The prevalence of LBP in both boys and girls increased as the grade increased, regardless of ECSA participation (Tables 1, 2). Although the ECSA group tended to have a higher prevalence of LBP relative to the non-ECSA group, this difference was significant in some but not all grades.

### Contribution of ECSA to LBP

In boys, the OR for ECSA-related LBP remained constant (1.02–1.24) from grade E4 to grade J2; however, it increased to 2.00 in grade J3 (95% CI: 1.47–2.71; *P* < 0.01) (Table 1). In girls, the ORs for ECSA-related LBP were 1.48 (95% CI: 1.00–2.20; *P* = 0.05) in grade E5 and 1.91 (95% CI: 1.33–2.75; *P* < 0.01) in grade E6 (Table 2); both ORs were significantly higher than those of boys in the same grades. The OR in girls was similar in grades J1 and J2 (1.2 and 1.23, respectively), but significantly increased in grade J3 (1.81; 95% CI: 1.26–2.61; *P* < 0.01).

The PAFs for ECSA-related LBP in boys were similar (10–16%) in all grades except E5 (1.1%). In contrast, the PAFs for girls varied from − 10.7 to 28.8%. Boys had a significantly higher PAF than girls in grade E4 (9.1% vs. -10.7%; *P* < 0.001). Conversely, girls had a significantly higher PAF than boys in grades E5 (19.0% vs. 1.1%, *P* < 0.001) and E6 (28.8% vs. 12.8%; *P* < 0.001).

## Discussion

We investigated the prevalence of LBP in boys and girls with particular consideration of ECSA. Our results clarified the similarities and differences in the association of LBP and ECSA between the genders.

Firstly, LBP was more prevalent in the ECSA group than in the non-ECSA group in almost every grade, regardless of gender; the difference was significant in some but not all grades. Moreover, the prevalence of LBP and the ORs were very high in both boys and girls in grade J3. Many students in Japan discontinue ECSA during the summer to prepare for high school entrance examinations in the next spring; hence, the J3 students in the ECSA group in our survey (which was conducted in the fall) are believed to be elite athletes. Elite athletes may have been practicing more intensely than ordinary athletes for a long time, which might contribute to the increased prevalence of LBP and relatively high ORs in grade J3 due to some mechanical factors, such as spondylolysis. Our previous cross-sectional study indicated that a history of LBP significantly increased in the group that spent a longer time participating in ECSA [18]. However, J3 students spent lesser time in ECSA with a higher prevalence of LBP than J2 students, suggesting that a high psychosocial burden both in school and during ECSA may play some roles in LBP among J3 students.

Previous studies have yielded different observations of the relationship between ECSA and LBP. Although some studies found no relationship between ECSA and LBP [23, 24], others reported a borderline relationship [3] or a relationship in the context of high physical activity [25]. Therefore, no conclusions have yet been drawn regarding ECSA as a risk factor for the development of LBP in children and adolescents [26]. Our current results strongly suggest that sports activities are linked to LBP in both boys and girls.

Secondly, the prevalence of LBP increased as the grade level increased, regardless of ECSA participation, in both boys and girls. This result is consistent with the findings of our previous cross-sectional studies [18, 20] and birth cohort analysis [19], although neither study had assessed gender-based differences between the ECSA and non-ECSA groups. It indicates that age-related factors in addition to sports activities promote LBP in both boys and girls.

Despite the aforementioned similarities between boys and girls, we also observed some differences. The ORs for ECSA-related LBP in grades E5 and E6 were considerably higher among girls than among boys. The PAF was also significantly higher in girls than in boys in both grades, although boys spent more time on ECSA. These results suggest that sports activities elicit different LBP-related responses in boys and girls, especially in the indicated grades.

Our study was unable to determine why girls were more vulnerable to ECSA-related LBP than boys in grades E5 and E6. However, another study on schoolchildren aged 9 to 15 years indicated that sports participants was significantly related to LBP only in girls, although boys undertook more strenuous activities [27]. Interestingly, they also showed that incidences of LBP in girls were much higher than in boys at 10 and 11 yo.

It was also reported that a high growth spurt is associated with increased LBP [7]. In Japanese girls, the time of peak height velocity occurs at an average age of 10.8 years (E5) [28]. The development of muscular strength is not accelerated during puberty in girls; however, it was accelerated throughout puberty in boys [29]. E5 and E6 are the grades in which many students start to exercise earnestly and the difference in muscular strength acceleration between boys and girls may explain why the incidence of ECSA-related LBP increased only in girls in grades E5 and E6.

Although among girls the prevalence of LBP was higher in grade J1 than in grade E6, both the OR and PAF were lower in grades J1 and J2. This finding suggests that LBP in grade J1 girls may be attributed to factors other than ECSA, including the onset of menstruation [30] (the average age of first menstruation in Japanese girls is 12 years) and psychogenic events, which occur more frequently in girls than boys [31].

The main strength of this study is its designation as the largest-scale birth cohort analysis in this field. The study data were derived from nearly the same groups of children and adolescents at each time point, which yielded more accurate results than those obtained in cross-sectional studies. However, this study also had several limitations. Although Niigata city is the 15th largest city in terms of population in Japan with both rural and urban areas and is considered a typical local city in Japan, the children in Niigata City might not necessarily be representative of Japanese children. Studies in other areas may be necessary to generalize the results in this study. The answers in the questionnaires were self-reported, and the students were not medically examined by a third party. Furthermore, this was not a true individual longitudinal study because the groups were not identical. Additionally, we were unable to examine other confounders, such as general health, possible diagnoses (e.g., spondylolysis), quality of life, family history, and psychological factors, which might have impacted the development of LBP. In particular, we think that mental health status is an important factor, even in children and adolescents; therefore, we have been preparing an investigation into the association between mental health and LBP in a separate study. Moreover, further longitudinal studies that track students individually with respect to physical and psychological factors are required.

## Conclusions

Although sports activities were associated with LBP in both boys and girls, they had a greater impact on girls, particularly those in grades E5 (ages 10–11 yo) and E6 (ages 11–12 yo). However, our study suggests that LBP in grades J1 and J2 girls were attributed to factors other than ECSA.

## Data Availability

Data are available on reasonable request from corresponding author.

## Abbreviations

CI: Confidence interval
E4–E6: Refer to elementary school grades 4–6
ECSA: Extracurricular sports activities
J1–J3: Refer to junior high school grades 1–3
LBP: Low back pain
OR: Odds ratio
PAF: Population attributable fraction
yo: years old
